# Validation of the Japanese version of MemScreen: a rapid screening tool for mild cognitive impairment

**DOI:** 10.1265/ehpm.25-00092

**Published:** 2025-12-03

**Authors:** Ai Ikeda, Hadrien Charvat, Takeshi Tanigawa, Nobuto Shibata, Koutatsu Maruyama, Kiyohide Tomooka, Yukari Asai, Juna Kamijima, Qisheng Li, Noemi Endo, Saori Miyazaki, Archana Singh-Manoux, Julien Dumurgier

**Affiliations:** 1 Department of Public Health, Graduate School of Medicine, Juntendo University, Tokyo, Japan; 2 Faculty of International Liberal Art, Juntendo University, Tokyo, Japan; 3 Mental Health Clinic, Juntendo Tokyo Koto Geriatric Medical Center, Tokyo, Japan; 4 Graduate School of Agriculture, Ehime University, Ehime, Japan; 5 Université Paris Cité, Inserm U1153, Epidemiology of Ageing & Neurodegenrative Diseases, Paris, France; 6 Faculty of Brain Sciences, University College London, London, UK

**Keywords:** Validation study, Screening, Mild cognitive impairment

## Abstract

This study was to examine the validity of the Japanese version of MemScreen (MemScreen-J), a touchscreen MCI screening test. 20 patients with MCI aged 65–90 years at the Juntendo Tokyo Koto Geriatric Medical Center were recruited as cases in December 2023. Non-cases were recruited from local residents in Toon City, Ehime Prefecture in February 2024 and 40 residents, without a medical history of MCI, aged 58–84 years were included in the present study. MemScreen-J test, a self-administered screening test in the form of a digital application, downloadable on a tablet, was administered to participants to assess their cognitive function. Defining the group at high risk of MCI based on a MemScreen-J test score of 28 or lower achieved the best Youden index in the study sample, with a sensitivity of 0.75 and a specificity of 0.98. MemScreen-J appeared to be a valid screening tool among persons at the prodromal stage of dementia, given reasonably high accuracy in detection of MCI. This innovative neuropsychological test could be the first step in a diagnostic approach to cognitive complaints in a community, identifying persons at the preclinical stage of dementia.

**Supplementary information:**

The online version contains supplementary material available at https://doi.org/10.1265/ehpm.25-00092.

## Introduction

Previous studies have reported that 50% of patients with mild cognitive impairment (MCI) will develop dementia within 5 years if left untreated [1]. The importance of early detection and intervention at an earlier stage– mild cognitive impairment (MCI) – is widely recognized, including the possibility that 40% of dementia might be prevented by improving lifestyle-related factors [2].

Much of the evidence on MCI is based on studies undertaken in specialized research centers, and instruments to assess MCI in community settings remain to be established. A good instrument needs to be easy to administer, not be too time consuming, and easily interpretable. These characteristics would allow assessment of MCI in multiple settings. In the present study, our aim was to test the validity of one such instrument, the Japanese version of MemScreen (MemScreen-J), a touchscreen MCI screening test developed by Université Paris Cité (Prof. Julien Dumurgier) [3, 4] and to use it in community settings.

## Methods

### Study design and population

MCI patients seen at the Juntendo Tokyo Koto Geriatric Medical Center in December 2023 were recruited on a voluntary basis and 20 patients aged 65–90 years were included as cases. These patients were diagnosed as having MCI using criteria defined by Petersen (2004) [5] by psychiatrists at the medical center. Non-cases were recruited from local residents in Toon City, Ehime Prefecture in February 2024. 40 residents aged 58–84 years volunteered to participate in the present study. Prior to inclusion in this study, medical history was used to exclude those with a previous diagnosis of MCI.

### Assessment of MemScreen-J

The MemScreen-J was developed via the back-translation method. The English version of MemScreen was translated into Japanese and then translated back into English by a native English speaker. The back-translation was undertaken in consultation with the developer of MemScreen, Prof. Dumurgier.

MemScreen-J, a self-administered screening test in the form of a digital application, downloadable on a tablet, was administered to cases and non-cases to assess their cognitive function. MemScreen-J is composed of a 12-point evaluation of verbal episodic memory (a list of 12 words), a task comprising a 5-point evaluation of temporal orientation, a 9-point test of calculation, a 4-point item on intruder recognition, and a 4-point clock reading. The MemScreen-J yields a 34-point overall score and the time taken to complete the test. Screenshots from the application are shown in Supplementary Fig. 1. MemScreen-J was administered in a private room for cases, and in a large room with soundproof protection in non-cases.

### Statistical analysis

T-test and chi-square tests were used to compare demographic characteristics including age (years), sex (male or female) and educational background (school attended until the age of ≤15 years or >15) in cases and non-cases. To examine the validity for the total score and total completion time of the MemScreen-J test, we constructed a Receiver Operating Characteristic (ROC) curve. The value maximizing the Youden index (sensitivity + specificity − 1) was chosen as to obtain optimal cut-off values for the MemScreen-J total score and completion time. We then constructed a logistic regression model for MCI status using the total score and the total completion time of MemScreen-J as predictors. All analyses were conducted using the SAS statistical package Version 9.1.

## Results

There was a statistically significant difference in age, but no differences were found in proportion of men and women or educational background when comparing MCI patients (cases) and the community residents (non-cases) (Table 1).

**Table 1:** Participant demographics.

	**MCI patients** **(n = 20)**	**The community** **residents (n = 40)**	**P-value**
Age, year (SD)	80.7 (6.80)	77.1 (4.85)	0.02
Male, n (%)	4 (20.0)	7 (17.5)	0.81
School attended until the age of <15 years, n (%)	6 (30.0)	6 (15.0)	0.17

**Fig. 1:**
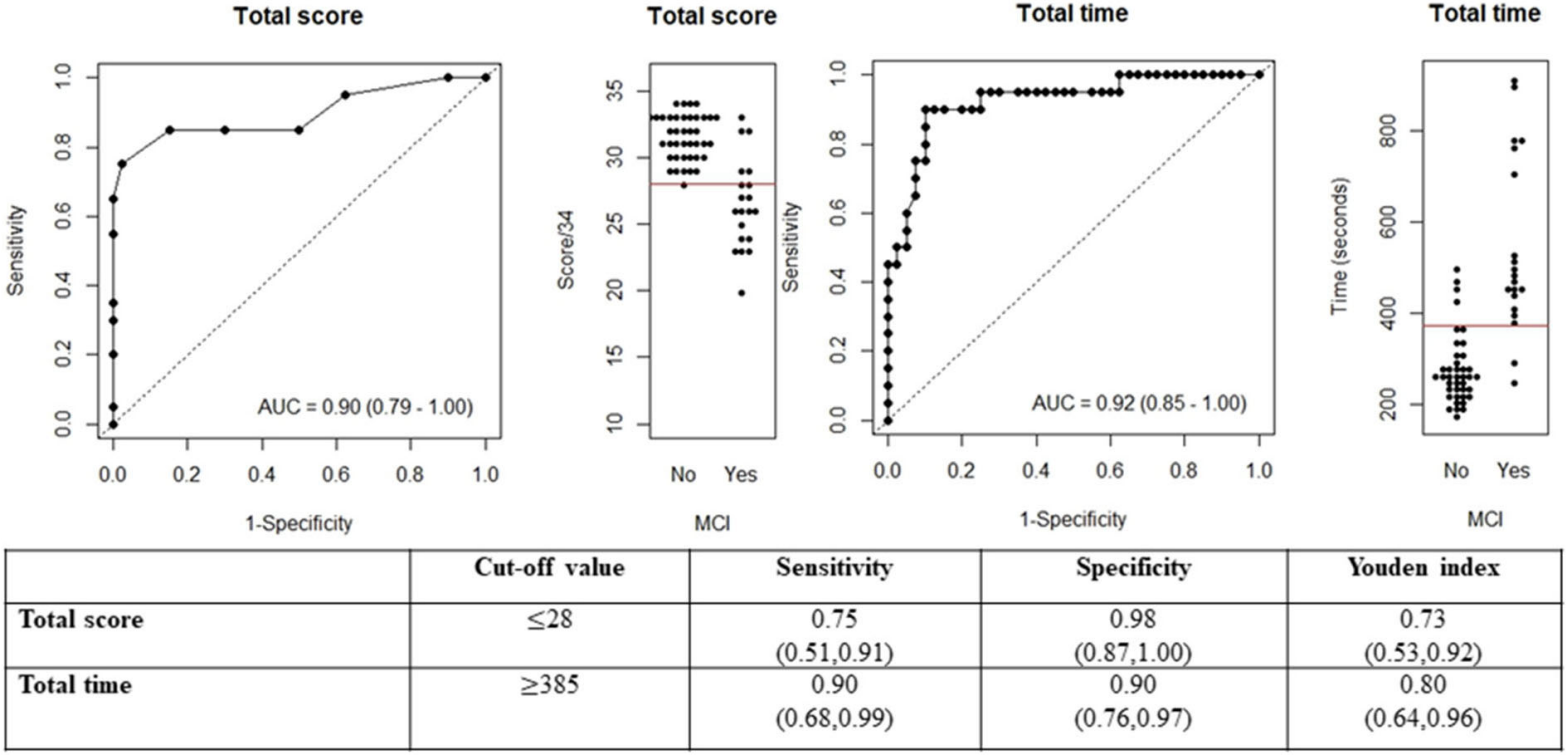
The value (95% confidence interval) of diagnostic accuracy measure for MemScreen-J test.

The mean total score of MemScreen-J was 26.6 (SD = 3.36) in MCI patients and 31.5 (SD = 1.68) in the community residents (data not tabulated). The total score of MemScreen-J was found to have excellent discrimination as the area under the ROC curve was 0.90 (Fig. 1). Defining the group at high risk of MCI based on a MemScreen-J test score of 28 or lower achieved the best Youden index in the study sample, with a sensitivity of 0.75 and a specificity of 0.98 (Fig. 1).

The mean total time taken to complete MemScreen-J was 541 seconds (SD = 195) in MCI patients and 275 seconds (SD = 78) in the community residents (data not tabulated). The total completion time of MemScreen-J also demonstrated exceptional discrimination, as the area under the ROC curve was 0.92 (Fig. 1). Defining the group as at high risk of MCI based on the total time of 385 seconds or longer achieved the best Youden index in the study sample, with a sensitivity of 0.90 and a specificity of 0.90 (Fig. 1).

The coefficients from logistic regression analysis with MemScreen-J total score and the total completion time as predictors and MCI as the outcome were used to construct the predicted risk as follows:

$$\begin{array}{rl} & \text{Model Predicted Risk}=\\ & \frac{1}{1+\exp(-9.9219102-0.01162518*\text{Time}+0.5076564*\text{Score})} \end{array}$$

**Fig. 2:**
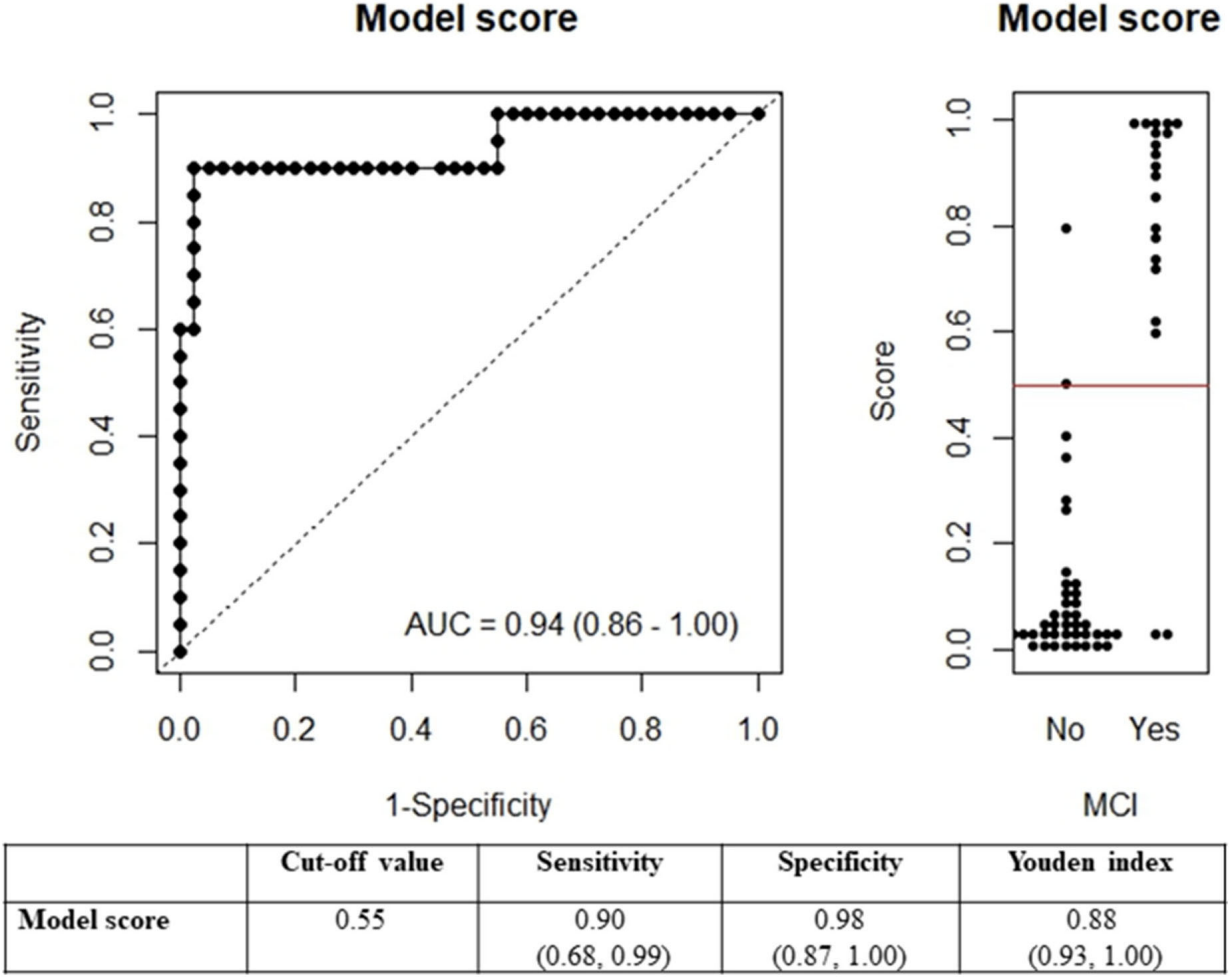
The predicted score and diagnostic accuracy measure (95% confidence interval) based on a logistic model.

A ROC curve (Fig. 2) based on predicted risk was constructed with various cut-off points for MCI classification. A cut-off predicted risk of 0.55, corresponding to an AUC of 0.94, had the best Youden index (0.88). The corresponding sensitivity was 0.90 and specificity was 0.98. As there was a significant difference in age between MCI patients and the community residents, we also fitted a model further adjusting for age. However, including age in the prediction model did not alter the performance of the MemScreen-J leading us not to retain age in the final analysis.

## Discussion

In the present study, MemScreen-J appeared to be a valid screening tool among persons at the preclinical stage of dementia, given its reasonably high accuracy in detection of MCI. We found fairly similar cut-offs for total score and total completion time based on the best Youden index, compared to those proposed in the previous study of French patients with amnestic syndrome [3].

A strength of this study is that this screening instrument provides a quick assessment of global cognitive function and the touchscreen tablet is simple to use. MemScreen-J does have the limitation that a normal result does not rule out early memory pathology. Indeed, this screening test is not designed to replace a specialized medical assessment of cognitive status. However, given the high specificity values for the MemScreen-J score and completion time of 0.98 and 0.90, respectively, obtained with the Youden index-based cut-off points, a normal test and the absence of other clinical features suggestive of progressive cognitive pathology will be reassuring for most individuals. Sensitivity values of 0.75 and 0.90 were obtained for MemScreen-J total score and completion time in the present study. In community settings, where most people are unlikely to have MCI [6], it is for screening tools to have a high degree of specificity in identifying possible cases of MCI, in order to avoid a large number of false positives. Although the total score of ≤28 had the best Youden index and was the optimal cut-off point to reduce misclassification in the identification of MCI in the risk prediction model in community settings in our study, the cut-offs may differ in other settings (i.e., high-risk settings). Moreover, the sample size in our study was small, but the event per variable (EPV) [7] was 20/1, suggesting that sample size was unlikely to bias logistic regression estimates. However, the interpretability of results in terms of sensitivity was limited, as reflected in the wide confidence interval. In addition, there may be potential bias introduced by differences in testing environments; however, given the substantial similarity in testing conditions between cases and non-cases, the impact of such bias on the findings is likely to be limited. Finally, as we did not specifically match cases and non-cases, there are likely to be other sources of differences in unmeasured confounding factors.

In conclusion, MemScreen-J appears to be a valid and simple screening tool for MCI. This innovative neuropsychological test could be the first step in a diagnostic approach to screening for cognitive status in community settings.

## Supplementary information

**Additional file 1:** **Supplementary Figure 1.** The selected screenshots from the MemScreen-J application.

## Declarations

### Availability of data and material

The data that support the findings of this study are available upon request from the corresponding author. The data are not publicly available due to privacy or ethical restrictions.
