# Supplementary figures and images for: Validation of the Japanese version of MemScreen: a rapid screening tool for mild cognitive impairment
Source: Environ Health Prev Med. 2025 Dec 3;30:96. doi: 10.1265/ehpm.25-00092 (PMC12698361; doi:10.1265/ehpm.25-00092)

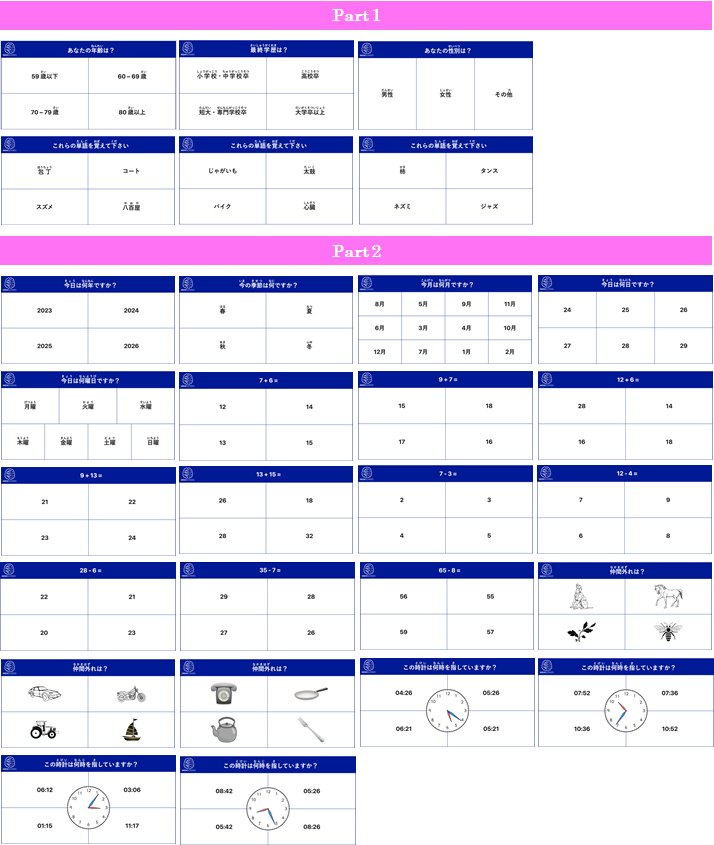

Supplement: Supplementary file 1 — Additional file 1: Supplementary Figure 1. The selected screenshots from the MemScreen-J application. [file ehpm-30-096-s001.tif]
